# Level I Hyperglycemia Alert: A Case Report

**DOI:** 10.5811/cpcem.2022.2.55160

**Published:** 2022-08-08

**Authors:** Michelle Nassal, Christopher San Miguel

**Affiliations:** The Ohio State University, Wexner Medical Center, Department of Emergency Medicine, Columbus, Ohio

**Keywords:** hyperglycemia, stroke mimic, nonketotic hyperglycemia

## Abstract

**Introduction:**

Nonketotic hyperglycemia-associated chorea is a rare condition that upon presentation to the emergency department can be easily misdiagnosed as a seizure or a stroke. Although uncommon, identification of this condition can aid emergency physicians in avoiding unnecessary and potentially harmful treatments for other neurological pathology. Furthermore, prompt hyperglycemic control can result in reversal of symptoms within days.

**Case Report:**

We present a case of nonketotic hyperglycemia-associated chorea where the patient was transferred to our facility as a hemorrhagic stroke alert, based on a false-positive interpretation of head computed tomography (CT) imaging.

**Conclusion:**

Nonketotic hyperglycemia on CT imaging and clinical presentation can mimic stroke presentations. Prompt recognition of key features can lead to appropriate treatment.

## INTRODUCTION

We present a case of a patient with nonketotic hyperglycemia hemichorea who was transferred to our facility as a hemorrhagic stroke alert. This alert was based on a hyperdensity seen on a non-contrast computed tomography (CT) of the head. Physical exam findings included choreiform movement of the patient’s right upper extremity. Magnetic resonance imaging (MRI) findings were consistent with nonketotic hyperglycemia hemichorea. The patient was admitted to medicine for diabetic control with improvement of her symptoms. She was ultimately discharged home several days later.

## CASE REPORT

A 57-year-old female with history of type 2 diabetes mellitus, coronary artery disease status post-coronary artery bypass graft, hypertension, hyperlipidemia, hypothyroidism, anxiety, and depression presented to the emergency department (ED). She reported five days of progressive, uncontrolled right arm movements and facial twitching that she could only briefly suppress. Her arm movements were also increasing in magnitude. On the day of her presentation, she also started to have “slurred speech” described as having difficulty enunciating words. She denied a prior history of traumatic brain injury, seizures, and/or stroke. Her home medications included insulin, aspirin, clopidogrel, risperidone, and venlafaxine, and no other relevant medications. The referring hospital read the patient’s head CT as an intracerebral hemorrhage and she was promptly transferred to our Comprehensive Stroke Center for further management.

Upon arrival to the ED, the patient’s vital signs were recorded as follows: blood pressure 117/58 millimeters of mercury, heart rate 77 beats per minute, temperature 36.7°C, saturating 94% on room air with respiratory rate 18 breaths per minute. Point-of-care glucose was 203 milligrams per deciliter (mg/dL) (reference range: 70–99 mg/dL). On physical exam, she was alert and oriented to person, place, and time. She did not have objective speech difficulties. She had choreiform movement of her right upper extremity and simultaneous right-sided facial grimacing that she could briefly suppress (Video1). She had right upper extremity ataxia and drift due to choreiform movement. She also exhibited right lower extremity drift. She denied any sensory deficits.

**Image 1:**
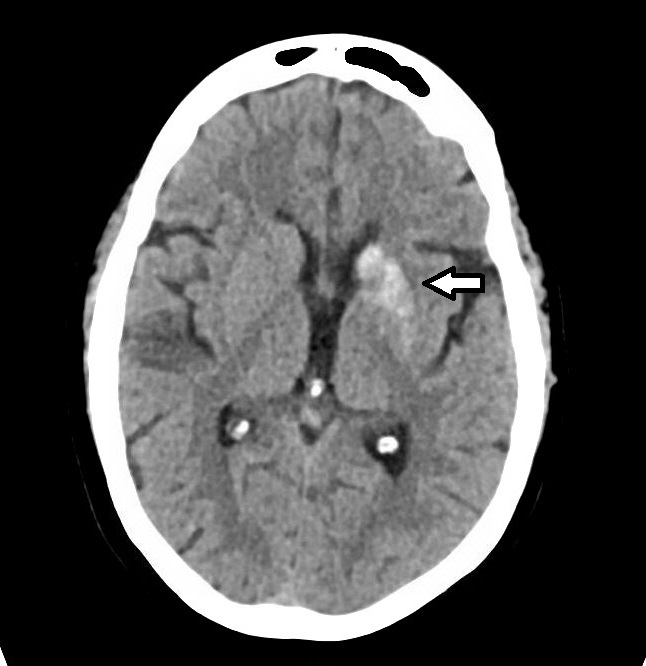
Computed tomography head imaging, which shows hyperintensity (arrow) in left caudate nucleus concerning for potential hemorrhagic stroke.

**Image 2:**
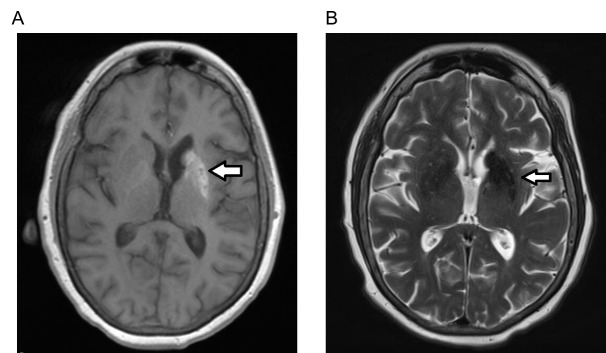
Magnetic resonance imaging of brain showing nonketotic hyperglycemia. (A) shows T1-weighted fluid-attenuated inversion-recovery (FLAIR) imaging with hyperintensity (arrow) in correlation with computed tomography imaging as seen Image 1. (B) on T2-FLAIR and diffusion-weighted imaging signal is hypointense (arrow) with no surrounding edema. This supports nonketotic hyperglycemia rather than acute hemorrhage.

A repeat head CT revealed a “hyperdensity within the left caudate and lentiform nucleus with apparent sparing of the anterior limb of the internal capsule” (Image 1). She also had a remote lacunar infarct near the left caudate. The neurovascular stroke team simultaneously evaluated the patient with the emergency physicians. As the patient’s exam was not consistent with a hemorrhagic stroke in the left caudate and lentiform nucleus, an emergent MRI of her brain was obtained. T1-weighted fluid-attenuated inversion recovery (FLAIR) hyperintense signal with T2-FLAIR and diffusion-weighted hypointense signal abnormalities without surrounding edema was observed on MRI. This was supportive of nonketotic hyperglycemia rather than acute hemorrhage (Image 2).

The patient’s laboratory findings were significant for hemoglobin A1c of 14.8% (4.7–5.6%) and negative urine ketones. She was admitted to a medicine service where she was followed by endocrinology and neurology. Her insulin regimen was increased, and carbohydrate-correction education was provided. After a four-day inpatient hospitalization, the patient’s chorea improved with decreased amplitude of the movements. The neurology team projected gradual improvement with improved glucose control. The patient was ultimately discharged home with advice for close follow-up.

## DISCUSSION

Nonketotic hyperglycemia-induced hemichorea (or hemiballismus) is a rare condition seen with uncontrolled diabetes. It is commonly initially misdiagnosed as a seizure due to the uncontrolled choreiform movement of one extremity.^1^ However, hemichorea can also be the presenting symptoms of an acute cerebrovascular accident in the basal ganglia, specifically in the subthalamic nucleus, or parietal lobe.^2^,^3^ Other diagnostic considerations include serotonin syndrome, neuroleptic malignant syndrome, post-streptococcal Sydenham chorea, hyperthyroid disorders, and tardive dyskinesia. The exact prevalence and pathophysiology of nonketotic hyperglycemia-induced hemichorea are unknown. Proposed causes include micro-ischemia to the basal ganglia from the hyperviscosity caused by hyperglycemia. Other proposed causes include interruptions in gamma-aminobutyric acid synthesis, micro-hemorrhagic injury, or auto-immune injury.^4^ Prompt identification of nonketotic hyperglycemia can lead to correct treatment with insulin and improvement in the hemichorea.

**CPC-EM Capsule**

What do we already know about this clinical entity?

*Nonketotic hyperglycemia-associated chorea is a rare condition that can mimic seizures and/or hemorrhagic stroke on computed tomography (CT)*.

What makes this presentation of disease reportable?

*Prompt identification can prevent potentially harmful hemorrhagic stroke interventions and facilitate correct treatment of glycemic management*.

What is the major learning point?

*Nonketotic hyperglycemia-associated chorea can mimic hemorrhagic stroke on CT, but prompt recognition can result in appropriate glycemic control*.

How might this improve emergency medicine practice?

*Awareness of this presentation can result in appropriate, emergent magnetic resonance imaging to confirm diagnosis and improve patient outcomes with appropriate treatment*.

Diagnostic tools to help identify nonketotic hyperglycemia-induced hemichorea include laboratory evaluation, CT and MRI. Surprisingly, this patient did not have significantly elevated glucose on presentation; however, her A1C was 14.8% suggesting poor diabetic control over the previous several months. Chronic poor diabetic control followed by several days of carefully controlled treatment to improve hemichorea suggests the basal ganglia insult is subacute. This raises the question: At which threshold of basal ganglia injury does a patient become acutely symptomatic? The hyperdensity seen on her CT was consistent with her ultimate diagnosis but could not be definitively distinguished from acute hemorrhage. To differentiate hemorrhagic stroke from nonketotic hyperglycemia MRI was necessary. According to Yu et al, MRI imaging, specifically differentiation on T1 and T2/FLAIR sequences, can help correctly identify nonketotic hyperglycemia.^5^ Emergent MRI confirmed nonketotic hyperglycemia. This allowed the patient to promptly begin carefully controlled diabetic management resulting in improvement of her symptoms. Without prompt identification, management may have been directed at treating a hemorrhagic stroke: antiepileptic medication, strict blood pressure control, reversal of anticoagulation, and potential neurosurgical procedures.

Nonketotic hyperglycemia hemichorea is rare. Within the last 20 years, an estimated 85 case reports have been published. mostly within neurologic journals.^6^–^8^ Due to its rarity, large databases with long-term clinical outcomes do not exist. However, small studies with four patients have shown that 50% of patients have long-term neurologic symptoms.^9^ Contrastingly, case reports have shown improvement, if not complete resolution, of chorea with continued diabetic control.^10^,^11^ Some case series (N = 12) have also suggested that the addition of dopamine receptor inhibitors and lorazepam can lead to neurological improvement in the majority of patients.^12^ This data suggests that clinical improvement with correct initial diagnosis and treatment is feasible, but a complete reversal of symptoms may not be achieved in all cases.

## CONCLUSION

Hemichorea presenting to the ED can be caused by several emergent diagnoses including acute cerebrovascular accident, seizures, and nonketotic hyperglycemia. Differentiation between these diagnoses requires laboratory studies with advanced imaging including CT and MRI. Knowledge of classic MRI radiologic findings is required to diagnose nonketotic hyperglycemic hemichorea. Appropriate early diagnosis of nonketotic hyperglycemia can lead to prompt treatment, improvement of symptoms, and costs savings without the harms of unnecessary interventions. Further, emergency physician awareness of imaging characteristics of nonketotic hyperglycemia hemichorea can lead to improved outcomes.

## Supplementary Information

Video Characteristic hemichorea and ipsilateral facial twitching of nonketotic hyperglycemia can be observed in this video.
